# Budget impact of wastewater surveillance for COVID-19 in a large urban jail: an activity-based cost analysis

**DOI:** 10.3389/fpubh.2026.1737517

**Published:** 2026-03-25

**Authors:** Victoria L. Phillips, Lindsay B. Saber, Shanika Kennedy, Matthew J. Akiyama, Anne C. Spaulding

**Affiliations:** 1 Department of Health Policy and Management, Rollins School of Public Health, Emory University, Atlanta, GA, United States; 2 Department of Global Health, Rollins School of Public Health, Emory University, Atlanta, GA, United States; 3 Department of Epidemiology, Rollins School of Public Health, Emory University, Atlanta, GA, United States; 4 Department of Medicine, Montefiore Hospital, New York, NY, United States

**Keywords:** budget impact analysis, correctional health, COVID-19, infectious disease preparedness, wastewater surveillance program

## Abstract

**Background:**

Correctional facilities were disproportionately affected by SARS-CoV-2 (COVID-19) outbreaks. Wastewater surveillance programs (WSP) may provide early warnings of the presence of COVID-19 and facilitate the timely implementation of infection mitigation, but their budgetary implications for correctional systems remain unclear.

**Methods:**

We conducted an activity-based costing analysis of a pilot WSP in a large urban jail. Costs borne by the jail were assessed and included start-up investments, implementation expenditures, and the costs of confirmatory polymerase chain reaction testing (PCR test). We developed budget impact scenarios based on the number of outflow points testing positive and the confirmatory testing required in the cellblocks linked to them. We also conducted a scenario analysis using rapid antigen tests in place of PCR tests.

**Results:**

Initial start-up costs totaled $29,471. Implementation costs for testing a single outflow point, linked to seven cellblocks, were $16,802, with PCR-testing accounting for 78% of expenditures. Implementation costs varied from $4,403 for zero positive outflow tests to $125,965 for eight positive outflow tests. Use of rapid antigen tests reduced costs significantly.

**Conclusion:**

Adoption of wastewater surveillance with confirmatory testing in correctional settings may entail substantial costs. The budget impact is likely to be highly sensitive to confirmatory test type, sewer configuration, and the specificity of the wastewater sample to a location. These findings underscore the need for correctional administrators to investigate feasibility and affordability when considering the use of WSP to help protect incarcerated populations from infectious disease threats.

## Background

Correctional facilities are disproportionately at risk for the spread of infectious disease outbreaks due to both the high-risk nature of congregate living and the challenges of implementing traditional public health measures. Early detection of outbreaks is critical for protecting both incarcerated individuals and staff, yet most correctional health systems lack scalable surveillance tools, a deficit underscored by the recent COVID-19 epidemic. Jail populations are particularly vulnerable to disease outbreaks given detainee turnover, within facility transfers, overcrowding, limited space for quarantine and isolation, and exposure from correctional staff (1–3).

At the outset of the COVID-19 pandemic, the Centers for Disease Control and Prevention (CDC) released specific guidelines for COVID-19 infection mitigation in correctional and detention facilities. These guidelines, however, noted that facility responses would depend on physical layouts, spatial constraints, healthcare service arrangements, and budgets, which could act as barriers to implementation (4).

During the COVID-19 pandemic, US correctional facilities generally tested detainees for COVID-19 on admission and isolated or quarantined based on nasal swab test (NST) results. Different strategies were used to identify cases among those currently in custody, including proactive diagnostic testing and/or reliance on symptom presentation of a detainee, with subsequent testing of people within the possible exposure areas (1). Case isolation and quarantine varied by facility and were highly dependent on space availability (5, 6).

Facilities used both rapid antigen tests and/or reverse transcription quantitative polymerase chain reaction (RT-qPCR) test (PCR-test) for case confirmation (1). The former is relatively low-cost and identifies transmissible infections in approximately 15 min, without the need for advanced laboratory testing equipment. Rapid antigen tests generally have high specificity but lower sensitivity than PCR tests, especially when viral load is low, increasing the risk of false-negative results (7). The PCR test has greater sensitivity; however, it also identifies cases that are no longer contagious, is more expensive, and generally requires a 24-h turnaround time from a laboratory (8).

While the properties of the tests do not differ across settings, jails introduce complexities in administering them that are not present in other congregate settings. These include restrictions on prisoner mobility and the need for correctional and medical staff to test within cellblocks, which can strain scarce security staff. Because of the frequently crowded nature of jails, and the higher risk of transmission, rapid tests are often used for convenience (9).

Research on the relative cost and benefits of rapid antigen vs. PCR testing is mixed and depends on setting, testing frequency, level of risk of transmission, and the outcome measure used. Rapid antigen testing was more cost-effective for limiting transmission in the outpatient setting, whereas PCR dominated in the hospital setting (10). Rapid antigen testing was found to be more cost-effective in terms of quality-adjusted life-years gained in homeless shelters, while in school settings, antigen testing with PCR follow-up was cost-effective compared to no testing during moderate outbreaks (11, 12). The relative costs and health benefits of the testing approaches in terms of preventing transmission in jails is unknown.

Wastewater surveillance programs (WSPs) emerged during the COVID-19 pandemic as an innovative strategy to monitor viral activity at the population level, once the SARS-CoV-2 RNA virus, the causative agent of COVID-19 infection, was shown to shed in fecal matter (13). Wastewater testing is highly accurate in detecting the presence or absence of disease. Predicting prevalence is difficult due to viral shedding rates, wastewater flow, and sample collection methods; however, several authors have addressed these issues through modeling and provide cases estimates based on viral load (14). By detecting SARS-CoV-2 RNA in sewage samples, WSPs can provide early warnings of infection clusters and guide targeted testing or isolation measures.

In congregate settings, such as jails, a positive test could potentially identify the presence of the virus in the specific area linked to the outflow point and facilitate targeted follow-up diagnostic testing to identify cases and limit outbreaks (15). Several studies confirmed the feasibility of wastewater surveillance. When coupled with PCR nasal swab testing specifically, the data indicated that COVID-19 cases could be identified up to 4 days before symptoms appeared, offering the possibility of limiting an outbreak (6, 15–22).

Wastewater surveillance is a non-invasive, anonymous, early warning surveillance tool for COVID-19 (23). WSPs are a passive surveillance measure and detect SARS-CoV-2 shedding from asymptomatic individuals who might not otherwise seek testing (24). Wastewater surveillance has several limitations. It can detect SARS-CoV-2 in wastewater up to 50 days after an individual tests positive via a nasal swab and is no longer infectious (15). The number of infected individuals cannot be determined from the sample, and sample interpretation is complicated by effluent travel time through the sewer system, non-human pollutants, and the fecal load (25).

Although wastewater surveillance has been piloted in universities, nursing homes, and community settings, its use in correctional facilities was limited. Prior to 2021, when the WSP examined here was implemented in the Fulton County Jail (FCJ), very few publications were available on wastewater-based surveillance in jails, although the literature has expanded since then (26, 27). Also, since its introduction with the onset of COVID-19, wastewater surveillance has become an increasingly used method for disease detection, for illnesses such as measles, Monkeypox, and Influenza in congregate settings, including some jails and prisons (28). Though less common, WSP can also be utilized for other detectable substances in wastewater, such as illicit drugs, though the number of publications is sparse (29).

Very few studies have assessed the economic resources requirements and budget impact of WSP implementation (30, 31). Several have examined aspects of costs, including sampling methods and comparing wastewater sampling to diagnostic testing alone, although in practice, the two must be used together to identify specific cases (32–34). Case studies of Nepal and Malawi provide a thorough description of implementation costs, but program start-up costs are omitted (35).

Correctional administrators face unique budgetary constraints and must weigh the feasibility of new interventions against competing priorities (36, 37). Our group conducted a feasibility study of a wastewater surveillance program from October 2021 to May 2022 in the Atlanta, FCJ. We found that samples from specified outflow points were significantly correlated with confirmed cases in the cell blocks linked to those outflow locations (38). Building on our feasibility study, we conducted a budget impact analysis of implementing a WSP, defined here as wastewater surveillance combined with nasal swab PCR testing, to inform decision-making and resource allocation in correctional health systems.

## Methods

### Setting

The FCJ is comprised of two residential towers with seven floors each. Each floor houses six cellblocks. Sewer piping runs vertically, linking seven cellblocks (one per floor) in stacks. Pipes from the two residential towers flow into eight sewer outflow points. Cellblocks house approximately 35 men, resulting in 245 men connected to each outflow point.

In brief, in the feasibility FCJ study, we mapped the FCJ sewer system as no comprehensive diagram was available. We used fluorescent dyes to track wastewater flow from the cell blocks to the jail's eight sewer outflow points. Once the mapping was complete, samples were collected weekly using Moore swabs: the swabs were suspended in the outflow points and collected the following day for processing (39). Samples were then sent to The Center for Global Safe WASH Laboratory of Emory University, where they were analyzed using quantitative RT-qPCR to test for the presence of SARS-CoV-2 (38).

A system for individual PCR diagnostic testing was established simultaneously. Staff supervised detainee self-administered nasal swabs in tubes imprinted with barcodes. Barcodes were scanned into the laboratory's online portal, pre-loaded with the facility's roster, where resident names were linked to a unique swab number. Swabs were shipped overnight to Northwell Laboratory in New York. Results were reported in the portal within 24 to 48 h, shared with the jail's medical staff, and recorded in the detainees' medical records.

### Data collection and analysis

We used an activity-based approach to estimate the cost and budget impact on the jail of undertaking a WSP, comprised of wastewater sampling and subsequent PCR-nasal swab testing. We reviewed project protocols and research staff visit notes before and during implementation and conducted interviews with jail staff to create an inventory of program inputs, including personnel, supplies, and equipment (40). Inputs were classified as start-up investments or those associated with ongoing implementation (41). Research-related activities were excluded from calculations. Input costs were valued in 2022 dollars.

For each input, we quantified units and priced them using market-based values or project budget information. To estimate labor costs, we identified hours per task and wages based on the job classification of the staff person who completed the task. We also used reports from other correctional facilities implementing similar programs to supplement estimates of start-up planning time and ongoing project coordination (42). Where multiple cost values for inputs were present, we used the average of the two values.

We multiplied units by price to estimate start-up costs, implementation costs, and total costs (41). We calculated the costs for a single sampling round of eight sewer outflow points and for confirmatory PCR nasal swab testing for a single cellblock of 35 men. We then extrapolated those values to estimate the testing costs associated with a positive SARS-CoV-2 signal from one sewer, which would initiate testing of 245 men. We then examined the budget impact as a function of the number of positive outflow points. We identified the percent of labor and PCR test costs as a portion of total program costs. We also conducted a sensitivity analysis to assess the cost implications of using a rapid antigen test instead of the PCR test.

### Wastewater surveillance inputs

#### Wastewater surveillance initial program planning

Personnel planning time, estimated at 7 h per staff member, was required at the outset to create a wastewater surveillance program. Planning staff included a senior jail administrator, the medical director, a correctional staff supervisor, an infection control nurse, the program manager, and a consultant to support planning.

#### Wastewater collection initial planning and training

Initiating wastewater sampling involved two key steps: mapping the sewer system and organizing and training staff for sample collection. Sewer maps were created by flushing fluorescent dye down toilets and tracking dye to the eight outflow points. We assumed the project manager spent 5 h training staff and planning logistics; that two senior jail staff and one maintenance staff person attended two 1-h planning meetings; and that a maintenance staff member conducted a 1-h training session for eight jail staff in the sewer mapping protocol.

We assumed 1 h of program manager time to organize each mapping. One person monitored each drainage point, and another, accompanied by a security escort, placed dyes in one toilet per cell block, six per floor, for seven floors. Mapping was supervised by the project manager and one maintenance person. We estimated 7 h to map each tower; each tower was mapped five times.

The program manager then developed sewer sampling protocols and coordinated with the WASH laboratory at an estimated 10 h per sampling round. We assumed that the maintenance staff provided a 2-h training session for the program manager on accessing and sampling the outflow points, after which the program manager trained eight staff.

#### Wastewater testing implementation

Moore's cotton swabs were suspended in the eight outflow points for ~24 h before recovery (39). We estimated 25 min total for sample collection. Samples were then transported to the WASH Laboratory. One hour of data management time was assumed per round.

### PCR diagnostic testing inputs

#### PCR diagnostic planning and training

PCR nasal swab testing included testing organization, staff training for collecting self-administered tests, data entry, and sample submission. We estimated 2 h of project manager time to prepare a training session and 2 h to conduct training sessions for six jail staff who supervise and conduct testing. Required equipment included one computer, a scanner, and one cart to support testing.

For each testing round, we assumed that the program manager spent 2 h organizing testing staff, collecting cellblock rosters, setting up testing materials, and determining test locations. A correctional staff supervisor was estimated to spend approximately 45 min arranging security officers to accompany staff, supported by 15 min of administrative staff time.

#### PCR diagnostic testing implementation

We assumed that three staff members and two officer escorts could collect samples from one cellblock of 35 detainees within 1 h. After collection, we estimated that test tube scanning and entry of personal information into the online portal would take 30 s per tube, and updating medical records based on test results would take 3 min per detainee.

## Results

Start-up costs for wastewater sampling were $25,186 (Table 1), with 99% of the total being labor-related. Program planning constituted 49% of costs, at $12,658, and sewer mapping, 39% at $11,473. Implementation costs for a round of wastewater sampling, defined as testing all eight outflow points, were $4,285, the majority of which, $4,200, were attributable to wastewater sample processing. Taken together, the total cost for wastewater surveillance was $29,471, with start-up costs comprising 85% of the total.

**Table 1:** Wastewater surveillance program start-up, implementation, and total costs.

**Inputs**	**Number of hours/units^a^**	**Cost per unit^b^ ($)**	**Total costs ($)**
Wastewater surveillance start-up^c^			
Program planning			
Senior managers^d^	35	334	11,704
Consultant	5	100	500
Program manager	10	45	454
Total			12,658
Wastewater sampling planning			
Program manager	16	45	727
Maintenance staff	2	21	42
Sampling staff	2	18	286
Total			1,055
Sewer mapping			
Planning for mapping			
Senior managers	2	334	1,338
Program manager	5	45	227
Maintenance staff	3	21	62
Officer escorts^e^	6	23	143
Total			1,770
Mapping implementation			
Program manager	85	45	3,862
Maintenance staff	70	21	1,453
Jail sampling staff	140	18	2,503
Officer escorts	70	23	1,613
Dye Packets	8	34	272
Total			9,703
Total start-up costs			25,186
Wastewater sampling implementation			
Sampling staff	4.05	18	73
Swabs	10	1.2	12
Moore's swab sample processing	8	525	4,200
Total			4,285
Wastewater surveillance program total			29,471

^a^Dye packets are cost per packet; swabs are cost per swab.

^b^Sources for unit costs are provided in Supplementary Appendix 1.

^c^Start-up costs are invariant to the number of sewer outflow points tested.

^d^Hourly wage (unit cost) is an average over all senior staff categories.

^e^Total hours reflect rounding.

The total cost for PCR nasal swab testing for a cellblock of 35 men was $3,146, broken down into $872 for start-up and $2,274 for implementation (Table 2). Completing PCR testing in a cellblock of 35 men cost $2,156, of which PCR nasal swab tests, including processing, totaled $2,013.

**Table 2:** PCR diagnostic testing start-up, implementation, and total costs.

**Input**	**Number of hours/units**	**Cost per unit/hour ($)^a^**	**Total cost^b^ ($)**
PCR testing start-up^c^			
Training			
Program manager	4	45	182
Jail staff	12	18	215
Supplies			
Computer	1	250	250
Scanners	1	105	105
Cart	1	120	120
Start-up Total			872
PCR nasal swab testing implementation			
Pre-testing organization^a^			
Program manager	2	45	91
Clerical staff	0.15	21	3
Correctional supervisor	0.45	52	24
Total			118
Testing			
Program manager	0.5	45	24
Testing staff	2	18	36
Officer escorts	2	23	46
Clerical staff	0.25	17.88	5
Medical records staff	1	21.75	22
PPE packet	1	10	10
Nasal swab processing	35	58	2,013
Total			2,156
Implementation total			2,274
PCR-testing total for one cellblock			3,146
One positive outflow point			
Number of PCR nasal swab tests for seven cellblocks			245
Start-up total^c^			872
Implementation total^d^			15,210
PCR testing total			16,802

^a^Sources for unit costs are available in Appendix 1.

^b^Units multiplied by unit costs and total costs may vary marginally due to rounding.

^c^Invariant to the number of cellblocks tested.

^d^Implementation costs: [$2,156 (cost per cellblock) ^*^7 (cellblocks)] +118 (testing set-up).

We estimated base-case costs for a positive SARS-CoV-2 test at one outflow point during a sampling round, which would trigger PCR diagnostic testing for the seven cell blocks of 35 men connected to that outflow point, for a total of 245 tests. Implementation testing costs under this scenario were $15,210, with the cost increase driven by those associated with the processing of PCR nasal swab tests. When start-up costs of $118 are accounted for, the total cost of follow-up PCR nasal swab testing is $16,082.

The total cost for establishing a WSP that combines wastewater surveillance and PCR diagnostic testing for a single positive outflow point was $42,900. This cost includes $26,098 for start-up and $16,082 for PCR-testing implementation.

To assess the budget impact, we varied the number of outflow points with a positive signal from zero to eight, leading to increases, primarily in associated diagnostic testing costs (Table 3). WSP costs ranged from 30,501 to 152,063. The rise in cost was due to additional nasal swab testing, which ranged from $4,403 to 125,965 as the number of outflow points increased. Labor's share of program costs fell from 86 to 22% as PCR test processing costs rose from zero to 74%. Eliminating sewer mapping from start-up expenses reduced WSP costs by $11,473.

**Table 3:** Wastewater surveillance program budget impact by number of positive outflow points.

**Cost**	**Number of outflow points testing positive for SARS-CoV-2**				
	**0**	**2**	**4**	**6**	**8**
Start-up^a^					
Wastewater sampling	25,226	25,226	25,226	25,226	25,226
PCR-testing	872	872	872	872	872
Total Start-up	26,098	26,098	26,098	26,098	26,098
Implementation					
Wastewater sampling^b^	4,285	4,285	4,285	4,285	4,285
PCR-testing^c^	118	30,420	60,840	91,260	121,680
Total implementation^d^	4,403	34,705	65,125	95,545	125,965
Total wastewater surveillance program costs	30,501	60,803	91,223	121,643	152,063
Personnel implementation costs as a percent of total implementation costs (%)	86	46	33	26	22
Percent of PCR-test processing (only) as a percent of total program cost (%)	0	46	62	69	74
Total Implementation costs with rapid antigen tests^e^	4,403	9,345	14,169	19,229	24,171

^a^Start-up costs are invariant to the number of outflow points tested.

^b^One round of wastewater testing includes all eight outflow points.

^c^We included one testing set-up cost regardless of the number of positive outflow points.

^d^Total implementation costs equal [$2,159 (cost per cellblock) ^*^ seven (cellblocks per outflow point)] + $118 (testing start-up).

^e^Total implementation costs, at $6 per test, equal [$373 (cost per cellblock) ^*^ seven (cellblocks per outflow point)] + $118 (testing start-up).

Rapid antigen tests cost $6 per test, compared to $58 per PCR test. If the rapid antigen test were used for confirmatory testing, total implementation costs would fall substantially. Diagnostic testing for a 35-man cellblock decreases from $2,013 to 373. For two positive outflow points, for example, testing costs fall from $30,420 under PCR testing to $4,942 ($353 ^*^ seven cellblocks ^*^ two towers) with antigen testing. Total implementation costs, including $872 start-up, range from $5,060 to 19,886.

## Discussion

This study presents an activity-based cost analysis of a pilot WSP in a large urban jail. By structuring results as budget scenarios, we provide practical information on start-up investments, implementation costs, and the financial implications under different testing strategies and positive test results. These findings aim to support correctional administrators and public health officials in evaluating the affordability and feasibility of WSP as part of pandemic preparedness and ongoing infection control.

Costs primarily reflect the structure of the FCJ's sewer system, in which outflow points identify very large areas of possible COVID-19 infection and are higher than would be observed if outflow points were linked to smaller, specific areas of the jail. Also, eliminating the need for sewer mapping reduced wastewater sampling start-up costs by 54%.

Our base case assumed that all men in cell blocks linked to a positive outflow point are PCR-tested. A sampling approach could be employed to reduce the number and cost of follow-up diagnostic testing. Savings could also be obtained by switching to rapid antigen testing. However, this must be balanced against the benefit of early detection of asymptomatic cases. It is also possible that the costs of PCR tests may decrease in the future, potentially reducing the savings from the antigen option.

Our estimates represent the expense of establishing a WSP and one round of sampling the eight outflow points. They do not include the costs associated with developing a comprehensive response plan, such as when and how sampling and testing should be repeated. The development of plans could facilitate their adoption. In a study based in Wales, correctional stakeholders expressed a lack of confidence in interpreting wastewater surveillance results and a concern over their ability to target specific locations. These issues could be addressed in assessments of the benefits of a WSP (42).

We stress that we provide only a cost description, not an assessment of the value of a WSP in reducing COVID-19 cases. Conducting a cost-effectiveness analysis would require comparing the additional costs of WSP and the possible gains from early detection with the costs and outcomes associated with the jail's standard operating procedures. These data were not available for comparison. Further, a complete assessment would need to project relative changes in morbidity and mortality from each approach. Data for this was also unavailable; it was beyond the scope of the study and has generally proven challenging to assess.

The numbers presented here should be viewed as estimates of the magnitude of spending rather than precise values, as they reflect several assumptions. The time to undertake activities was based on reported task performance by research staff and retrospective reports from jail staff, which may have introduced bias. Research staff may have been able to perform tasks more quickly, such as testing per cellblock, compared to correctional staff. Time estimates may be greater if research-related tasks were not excluded from estimates (40). Program efficiencies may develop over time, thereby reducing ongoing labor costs.

Other assumptions included that: no sample wastage and that samples were accurate; that detainees were willing to continue testing; that software for uploading nasal swab test sample data worked; and that Internet access was consistently available. Estimates of mapping costs should be viewed with caution. The research team repeated the exercise numerous times to understand the flow of the sewer system, prior to obtaining facility blueprints, after the initial study had ended. Mapping procedures were influenced by the availability of security escorts. Further, if sewer plans had been available at the outset, mapping may have been unnecessary. Eliminating the need for mapping would have reduced WSP start-up costs by 46%.

We also assumed that the jail would have staff available to support WSP activities. During the pandemic, however, facilities faced significant operational challenges, suggesting substantial limits on staff's ability to redirect their efforts (43). Estimates of management planning time, based on the literature, are likely conservative. Four sites participating in a pilot study of a wastewater surveillance program withdrew due to organizational burden (44).

Ongoing vacancies among correctional staff also undermine the assumption of staff availability. Vacancy rates in Georgia have been estimated to average 50% and were exacerbated by the pandemic (45, 46). The cost of securing additional staff, if needed, has not been included in our calculations. During the pandemic, officers also reported challenges related to ongoing policy changes within the jail, which may influence WSP adoption (43).

To date, WSP have focused on limiting the spread of infection among detainees by testing detainees. One factor that has yet to be addressed is the role of correctional officers in disease transmission. At the FCJ, correctional officers underwent voluntary testing and were to isolate or quarantine based on test results, but testing was not mandatory. Guards moving throughout the jails and passing in and out of communities must be addressed as part of any infection control plan (46, 47).

This study is among the first to provide a detailed, activity-based accounting of the costs of implementing wastewater surveillance for SARS-CoV-2 in a correctional setting. By explicitly structuring results as budget scenarios, we highlight the financial implications of adopting WSP for the FCJ, which are highly dependent on the number of outflow points testing positive and the use of PCR diagnostic testing. For decision-makers, this variability underscores the importance of planning for extreme values and examining where efficiencies could be gained.

Framing WSP adoption as a budget impact analysis offers practical insights into decision-making around its adoption. Correctional administrators can use this method to forecast annual expenditures, justify funding requests, and weigh wastewater testing against alternative outbreak-control measures. Importantly, the financial case for WSP should also be viewed alongside its potential to prevent costly outbreaks, hospitalizations, and operational disruptions.

The public health implications here are significant. Correctional facilities remain vulnerable to infectious disease outbreaks, and developing pandemic preparedness plans is critical for the wellbeing of detainees and correctional staff (24). By clarifying the resource requirements of WSP, this study provides a foundation for realistic budget planning. It can inform broader debates about scaling wastewater surveillance to protect high-risk, marginalized populations and about its use for surveillance of other conditions.

## Data Availability

Data will be made available by the authors, upon request.

## References

[1] Hagan LM McCormick DW Lee C Sleweon S Nicolae L Dixon T . Outbreak of SARS-CoV-2 B.1.617.2 (Delta) variant infections among incarcerated persons in a federal prison—Texas, July–August 2021. MMWR Morb Mortal Wkly Rep. (2021) 70:1349–54. doi: 10.15585/mmwr.mm7038e3 PMC8459894 34555009

[2] Kirbiyik U Binder AM Ghinai I Zawitz C Levin R Samala U . Network characteristics and visualization of COVID-19 outbreak in a large detention facility in the United States—Cook County, Illinois, 2020. MMWR Morb Mortal Wkly Rep. (2020) 69:1625–30. doi: 10.15585/mmwr.mm6944a3 PMC7643900 33151915

[3] Leibowitz AI Siedner MJ Tsai AC Mohareb AM. Association between prison crowding and COVID-19 incidence rates in Massachusetts prisons, April 2020–January 2021. JAMA Intern Med. (2021) 181:1315–21. doi: 10.1001/jamainternmed.2021.4392 34369964 PMC8353573

[4] National Center for Immunization and Respiratory Diseases (US) Division Division of Viral Diseases. Interim Guidance on Management of Coronavirus Disease 2019 (COVID-19) in Correctional and Detention Facilities. Atlanta, GA: Centers for Disease Control and Prevention (2020).

[5] Beaudry G Zhong S Whiting D Javid B Frater J Fazel S. Managing outbreaks of highly contagious diseases in prisons: a systematic review. BMJ Glob Health. (2020) 5:e003201. doi: 10.1136/bmjgh-2020-003201 33199278 PMC7670855

[6] Puglisi LB Brinkley-Rubinstein L Wang EA. COVID-19 in carceral systems: a review. Annu Rev Criminol. (2023) 6:399–422. doi: 10.1146/annurev-criminol-030521-103146

[7] Sabat J Subhadra S Rath S Ho LM Satpathy T Pattnaik D . A comparison of SARS-CoV-2 rapid antigen testing with real-time RT-PCR among symptomatic and asymptomatic individuals. BMC Infect Dis. (2023) 23:87. doi: 10.1186/s12879-022-07969-0 PMC9909630 36759762

[8] Smith-Jeffcoat SE Mellis AM Grijalva CG Talbot HK Schmitz J Lutrick K . SARS-CoV-2 viral shedding and rapid antigen test performance — respiratory virus transmission network, November 2022–May 2023. MMWR Morb Mortal Wkly Rep. (2024) 73:365–71. doi: 10.15585/mmwr.mm7316a2 38668391 PMC11065460

[9] Lee HY Park YJ Lee SE Lee JJ Choi J Yu M . Preventing COVID-19 in correctional facilities: the impact of rapid antigen tests on early detection and infection control. Infect Chemother. (2025) 57:321–3. doi: 10.3947/ic.2024.0080 40490390 PMC12230384

[10] Kendall EA Arinaminpathy N Sacks JA Manabe YC Dittrich S Schumacher SG . Antigen-based rapid diagnostic testing or alternatives for diagnosis of symptomatic COVID-19: a simulation-based net benefit analysis. Epidemiology. (2021) 32:811–9. doi: 10.1097/EDE.0000000000001400 34292212 PMC8478097

[11] Cox SN Chow EJ Rolfes MA Mosites E Sharma M Chu HY . Health and economic impact of COVID-19 surveillance testing in Seattle homeless shelters: a cost-effectiveness analysis. AJPM Focus. (2025) 4:100307. doi: 10.1016/j.focus.2024.100307 40061153 PMC11889550

[12] Maya S McCorvie R Jacobson K Shete PB Bardach N Kahn JG. COVID-19 testing strategies for K-12 schools in California: a cost-effectiveness analysis. Int J Environ Res Public Health. (2022) 19:9371. doi: 10.3390/ijerph19159371 35954728 PMC9367893

[13] Kitajima M Ahmed W Bibby K Carducci A Gerba CP Hamilton KA . SARS-CoV-2 in wastewater: state of the knowledge and research needs. Sci Total Environ. (2020) 739:139076. doi: 10.1016/j.scitotenv.2020.139076 32758929 PMC7191289

[14] Haraguchi M Klaassen F Cohen T Salomon JA Menzies NA. Statistical relationship between wastewater data and case notifications for COVID-19 surveillance in the United States from 2020 to 2023: Bayesian hierarchical modeling approach. JMIR Public Health Surveill. (2025) 11:e68213. doi: 10.2196/68213 40402554 PMC12121544

[15] Wang Y Liu P Zhang H Ibaraki M VanTassell J Geith K . Early warning of a COVID-19 surge on a university campus based on wastewater surveillance for SARS-CoV-2 at residence halls. Sci Total Environ. (2022) 821:153291. doi: 10.1016/j.scitotenv.2022.153291 35090922 PMC8788089

[16] Hagan LM Williams SP Spaulding AC Toblin RL Figlenski J Ocampo J . Mass testing for SARS-CoV-2 in 16 prisons and jails — six jurisdictions, United States, April–May 2020. MMWR Morb Mortal Wkly Rep. (2020) 69:1139–43. doi: 10.15585/mmwr.mm6933a3 32817597 PMC7439979

[17] Njuguna H Wallace M Simonson S Tobolowsky FA James AE Bordelon K . Serial laboratory testing for SARS-CoV-2 infection among incarcerated and detained persons in a correctional and detention facility — Louisiana, April–May 2020. MMWR Morb Mortal Wkly Rep. (2020) 69:836–40. doi: 10.15585/mmwr.mm6926e2 32614816 PMC7332096

[18] Tompkins LK Gunn JKL Cherney B Ham JE Horth R Rossetti R . Mass SARS-CoV-2 testing in a dormitory-style correctional facility in Arkansas. Am J Public Health. (2021) 111:907–16. doi: 10.2105/AJPH.2020.306117 33734845 PMC8033997

[19] Tsoungui Obama HCJ Adil Mahmoud Yousif N Alawam Nemer L Ngougoue Ngougoue PM Ngwa GA Teboh-Ewungkem M . Preventing COVID-19 spread in closed facilities by regular testing of employees: an efficient intervention in long-term care facilities and prisons? PLoS One. (2021) 16:e0249588. doi: 10.1371/journal.pone.0249588 PMC8062045 33886605

[20] Zawitz C Welbel S Ghinai I Mennella C Levin R Samala U . Outbreak of COVID-19 and interventions in a large jail — Cook County, Illinois, United States, 2020. Am J Infect Control. (2021) 49:1129–35. doi: 10.1016/j.ajic.2021.03.020 PMC8016534 33813042

[21] Betancourt WQ Schmitz BW Innes GK Prasek SM Pogreba Brown KM Stark ER . COVID-19 containment on a college campus via wastewater epidemiology, targeted clinical testing, and an intervention. Sci Total Environ. (2021) 779:146408. doi: 10.1016/j.scitotenv.2021.146408 PMC7954642 33743467

[22] Park SK Lee CW Park DI Woo HY Cheong HS Shin HC . Detection of SARS-CoV-2 in fecal samples from patients with asymptomatic and mild COVID-19 in Korea. Clin Gastroenterol Hepatol. (2021) 19:1387–94.e2. doi: 10.1016/j.cgh.2020.06.005 32534042 PMC7286243

[23] Shah S Gwee SXW Ng JQX Lau N Koh J Pang J. Wastewater surveillance to infer COVID-19 transmission: a systematic review. Sci Total Environ. (2022) 804:150060. doi: 10.1016/j.scitotenv.2021.150060 34798721 PMC8423771

[24] Centers for Disease Control and Prevention. Targeted Wastewater Surveillance at Facilities and Institutions. Atlanta, GA: CDC.

[25] Wong MC Huang J Lai C Ng R Chan FKL Chan PKS. Detection of SARS-CoV-2 RNA in fecal specimens of patients with confirmed COVID-19: a meta-analysis. J Infect. (2020) 81:e31–8. doi: 10.1016/j.jinf.2020.06.012 32535156 PMC7289116

[26] Kennedy S Spaulding AC Surveillance Working Group. Four models of wastewater surveillance for SARS-CoV-2 in jail settings: how monitoring wastewater complements individual screening. medRxiv [Preprint]. (2023). doi: 10.1101/2023.08.04.23293152

[27] Saloner B Kramer C Song M Doan B Eber GB Rubenstein LS . COVID-19 restrictions in jails and prisons: perspectives from carceral leaders. Health Aff (Millwood). (2023) 42:841–8. doi: 10.1377/hlthaff.2022.01473 37276483

[28] Centers for Disease Control and Prevention. National Wastewater Surveillance System (NWSS). Atlanta (GA): CDC.

[29] Egaña I Nogales-Garcia M Akhrimenko V Gónzalez-Gómez X Quintana JB Villanueva-Blasco VJ . Wastewater-based epidemiology for the surveillance of illicit drug and substance use in prison settings: a critical review. WIREs Forensic Sci. (2025) 7:e70004. doi: 10.1002/wfs2.70004

[30] Harris-Lovett S Nelson KL Beamer P Bischel HN Bivins A Bruder A . Wastewater surveillance for SARS-CoV-2 on college campuses: initial efforts, lessons learned, and research needs. Int J Environ Res Public Health. (2021) 18:4455. doi: 10.3390/ijerph18094455 33922263 PMC8122720

[31] Maryam S Ul Haq I Yahya G Ul Haq M Algammal AM Saber S . COVID-19 surveillance in wastewater: an epidemiological tool for the monitoring of SARS-CoV-2. Front Cell Infect Microbiol. (2022) 12:978643. doi: 10.3389/fcimb.2022.978643 36683701 PMC9854263

[32] Liu P Ibaraki M VanTassell J Geith K Cavallo M Kann R . A sensitive, simple, and low-cost method for COVID-19 wastewater surveillance at an institutional level. Sci Total Environ. (2022) 807(Pt 3):151047. doi: 10.1016/j.scitotenv.2021.151047 34673061 PMC8522675

[33] Hart OE Halden RU. Computational analysis of SARS-CoV-2/COVID-19 surveillance by wastewater epidemiology locally and globally: feasibility, economy, opportunities and challenges. Sci Total Environ. (2020) 730:138875. doi: 10.1016/j.scitotenv.2020.138875 PMC7175865 32371231

[34] Wright J Driver EM Bowes DA Johnston B Halden RU. Comparison of high-frequency in-pipe SARS-CoV-2 wastewater surveillance to concurrent COVID-19 random clinical testing on a public U. S university campus. Sci Total Environ. (2022) 820:152877. doi: 10.1016/j.scitotenv.2021.152877 PMC8732902 34998780

[35] Ngwira LG Sharma B Shrestha KB Dahal S Tuladhar R Manthalu G . Cost of wastewater environmental surveillance for SARS-CoV-2: evidence from pilot sites in Blantyre, Malawi and Kathmandu, Nepal. PLOS Glob Public Health. (2022) 2:e0001377. doi: 10.1371/journal.pgph.0001377 PMC10021894 36962924

[36] Water Environment Federation. Pilot Program for Onsite Testing of SARS-CoV-2 in Correctional Facility Wastewater. Alexandria (VA): WEF. (2022).

[37] González-Montalvo MDM Dickson PF Saber LB Boehm RA Phillips VL Akiyama MJ . Opinions of former jail residents about self-collection of SARS-CoV-2 specimens paired with wastewater surveillance: a qualitative study. PLoS ONE. (2023) 18:e0285364. doi: 10.1371/journal.pone.0285364 PMC10166543 37155633

[38] Saber LB Kennedy SS Yang Y Moore KN Wang Y Hilton SP . Correlation of SARS-CoV-2 in wastewater and individual testing results in a jail, Atlanta, Georgia, USA. Emerg Infect Dis. (2024) 30:S21–7. doi: 10.3201/eid3013.230775 38561638 PMC10986836

[39] Sikorski MJ Levine MM. Reviving the “Moore swab”: a classic environmental surveillance tool involving filtration of flowing surface water and sewage water to recover typhoidal Salmonella bacteria. Appl Environ Microbiol. (2020) 86:e00060–20. doi: 10.1128/AEM.00060-20 32332133 PMC7301852

[40] Huebschmann AG Trinkley KE Gritz M Glasgow RE. Pragmatic considerations and approaches for measuring staff time as an implementation cost in health systems and clinics. Implement Sci Commun. (2022) 3:44. doi: 10.1186/s43058-022-00292-4 PMC9013046 35428326

[41] Haddix AC Teutsch SM Corso PS. Prevention Effectiveness: a Guide to Decision Analysis and Economic Evaluation. 2nd Edn. Oxford: Oxford University Press. (2003). doi: 10.1093/oso/9780195148978.001.0001

[42] Jones G Nelson A Chadwick DR Cobley S Jones DL Perrett S . Evaluation of wastewater surveillance for SARS-CoV-2 in a prison population: a mixed-methods approach. Front Public Health. (2024) 12:1462186. doi: 10.3389/fpubh.2024.1462186 39628802 PMC11611585

[43] Novisky MA Tostlebe J Pyrooz D Sanchez JA. The COVID-19 pandemic and operational challenges, impacts, and lessons learned: a multi-methods study of U. S prison systems. Health Justice. (2023) 11:51. doi: 10.1186/s40352-023-00253-6 PMC10696818 38051375

[44] Gondles E Miskell M Beyene M. Staff recruitment and retention in corrections. Correct Today. 2023;37–47.

[45] Prabhu MT. Nearly half of Georgia corrections officers' positions vacant. Atlanta J-Const. (2024).

[46] Nowotny KM Seide K Brinkley-Rubinstein L. Risk of COVID-19 infection among prison staff in the United States. BMC Public Health. (2021) 21:1036. doi: 10.1186/s12889-021-11077-0 34078350 PMC8170443

[47] Toblin RL Cohen SI Hagan LM. SARS-CoV-2 infection among correctional staff in the federal bureau of prisons. Am J Public Health. (2021) 111:1164–7. doi: 10.2105/AJPH.2021.306237 33856883 PMC8101570

