# Supplementary material for: Budget impact of wastewater surveillance for COVID-19 in a large urban jail: an activity-based cost analysis
Source: Front Public Health. 2026 Mar 25;14:1737517. doi: 10.3389/fpubh.2026.1737517 (PMC13057488; doi:10.3389/fpubh.2026.1737517)
Supplement: Supplementary file 1 [file Table_1.docx]

Appendix 1: Unit Cost Sources

| Resource Input | Unit Cost | Source |
| --- | --- | --- |
| *Personnel* |  |  |
| Senior administrator | 24 | https://www.salary.com/research/salary/hiring/jail-administrator-salary?pay=Hour |
| Medical director | 147 | https://www.salary.com/tools/salary-calculator/Associate-Medical-Director-hourly |
| Correctional supervisor | 52 | https://www.federalpay.org/gs/2022/georgia |
| Infection control nurse | 49 | https://www.salary.com/tools/salary-calculator/Nurse-Infection-Control-hourly |
| Healthcare Consultant | 36 | https://www.salary.com/research/salary/recruiting/healthcare-consultant-salary |
| Program manager | 45 | https://www.bls.gov/oes/current/oes131082.htm |
| Maintenance staff | 21 | https://www.bls.gov/ooh/installation-maintenance-and-repair/general-maintenance-and-repair-workers.htm |
| Officer escort | 23 | https://www.bls.gov/ooh/protective-service/correctional-officers.htm#:~:text=Pay%20About%20this%20section&text=more%20than%20%2478%2C330.-,The%20median%20annual%20wage%20for%20correctional%20officers%20and%20jailers%20was,percent%20earned%20more%20than%20%2479%2C340. |
| Sampling staff | 18 | https://www.salary.com/tools/salary-calculator/Medical-Assistant-Non-Certified-hourly |
| Jail staff | 18 | https://www.salary.com/research/salary/hiring/jail-clerk-salary?pay=Hour |
| Medical records staff | 22 | https://www.ziprecruiter.com/Salaries/Medical-Records-Clerk-Salary-in-Atlanta,GA |
| Clerical Staff | 17.88 | https://www.ziprecruiter.com/Salaries/Clerical-Staff-Salary |
| *Supplies* |  |  |
| Computer | 250 | https://www.amazon.com/HP-Performance-Laptop-14-LED-Backlit/dp/B07M7JM2XJ/ref=sr_1_15?crid=USRM1GAR3YGY&keywords=laptop+with+ethernet+port&qid=1658248114&s=electronics&sprefix=laptop+with+ethern%2Celectronics%2C329&sr=1-15 |
| Cart | 120 | https://www.amazon.com/Olympia-Tools-85-188-Collapsible-Service/dp/B00M0V5PWA/ref=sr_1_1_sspa?keywords=foldable+carts&qid=1658255227&sr=8-1-spons&psc=1&spLa=ZW5jcnlwdGVkUXVhbGlmaWVyPUFJOE1TN1lVTlBZSk8mZW5jcnlwdGVkSWQ9QTAyNjk3MzgxRVBMUUZZNEtIREUyJmVuY3J5cHRlZEFkSWQ9QTA4MjE0NDRSQVE3NzlDVldFNEsmd2lkZ2V0TmFtZT1zcF9hdGYmYWN0aW9uPWNsaWNrUmVkaXJlY3QmZG9Ob3RMb2dDbGljaz10cnVl |
| Scanner | 105 | https://www.amazon.com/WoneNice-Barcode-Scanner-Handheld-Reader/dp/B00LE5VV1C/ref=sr_1_3?crid=30H6B1PHCLU2W&keywords=barcode+scanners&qid=1658248195&s=electronics&sprefix=barcode+scanner%2Celectronics%2C124&sr=1-3 |
| Dye | 8 |  |
| Swabs for wastewater collection | 10 | Personal quote from? |
| Moore’s swab sample processing for eight outflow points | 525 | Average of prices quoted from Biobot labs and the Center for Global Safe WASH Laboratory of Emory University |
| Nasal swab PCR testing processing | 58 | Median price from personal communication with three sample processing labs. |
